# Beyond Glycemic Control: Ocular Effects of Glucagon-like Peptide-1 Receptor Agonists

**DOI:** 10.3390/vision10020029

**Published:** 2026-05-14

**Authors:** Filippo Lixi, Mario Troisi, Valerio Calabresi, Anina Giagoni, Costanza Rossi, Mihaela-Madalina Timofte-Zorila, Tudor-Corneliu Tarași, Livio Vitiello, Mara-Ioana Tomi, Alina-Gabriela Gheorghe, Giulia Coco, Giulia Lanzolla, Giuseppe Giannaccare

**Affiliations:** 1 Eye Clinic, Department of Surgical Sciences, University of Cagliari, Via Università 40, 09124 Cagliari, Italyvalerio.calabresi1@gmail.com (V.C.); giagonianina@gmail.com (A.G.);; 2 Ophthalmology Unit, Salerno University Hospital, Via San Leonardo, 84131 Salerno, Italy; 3 Department of Ophthalmology, Grigore T. Popa University of Medicine and Pharmacy, 700115 Iasi, Romaniatarasitudor@gmail.com (T.-C.T.); 4 Department of Ophthalmology, Cai Ferate Clinical Hospital, 700506 Iasi, Romania; 5 Eye Unit, Luigi Curto Hospital, Azienda Sanitaria Locale Salerno, 84035 Polla, Italy; livio.vitiello@gmail.com; 6 Department of Ophthalmology, Clinical Institute of Ophthalmological Emergencies “Prof. Dr. Mircea Olteanu”, 010464 Bucharest, Romania; 7 Ophthalmology Unit, Department of Clinical Sciences and Translational Medicine, University of Rome Tor Vergata, 00133 Rome, Italy; 8 Endocrinology Unit, Department of Medical Sciences, University of Cagliari, 09042 Monserrato, Italy

**Keywords:** GLP-1 receptor agonists, incretin therapy, diabetic retinopathy, NAION, age-related macular degeneration, glaucoma, dry eye disease, cataract, retinal vascular occlusion, ocular safety

## Abstract

Glucagon-like peptide-1 receptor agonists (GLP-1RAs) and newer dual-incretin therapies have become central to the treatment of diabetes mellitus and obesity, with benefits extending beyond glycemic control. Their expanding use has prompted growing interest in their potential ocular effects. Experimental data support plausible protective mechanisms, including reduction in oxidative stress and neuroprotective effects on retinal and optic nerve tissues. Clinical evidence, however, remains heterogeneous. In diabetic retinopathy, the main concern appears to be transient early worsening associated with rapid glycemic improvement rather than direct retinal toxicity. A potential semaglutide-associated signal for non-arteritic anterior ischemic optic neuropathy has raised concern, although the absolute risk appears low and causality remains unproven. Emerging studies also suggest possible beneficial associations with glaucoma, ocular surface diseases, and certain retinal vascular outcomes, whereas the evidence regarding age-related macular degeneration and cataract remains conflicting or preliminary. Overall, ocular outcomes associated with incretin-based therapies seem to reflect a complex interplay among drug-specific pharmacology, systemic metabolic changes, and individual patient susceptibility rather than a class effect. Baseline ophthalmic assessment and individualized follow-up may be advisable in selected high-risk patients. Further prospective ophthalmology-focused studies are needed to clarify long-term safety and identify the patients most likely to benefit or develop adverse events.

## 1. Introduction

Glucagon-like peptide-1 receptor agonists (GLP-1RAs) have emerged as a cornerstone of modern metabolic therapy, with established efficacy in type 2 diabetes mellitus (T2DM), obesity, and cardiometabolic disease [1]. Originally developed to exploit the physiological incretin effect, these agents enhance glucose-dependent insulin secretion, suppress glucagon release, delay gastric emptying, and promote satiety, thereby improving glycemic control while minimizing the risk of hypoglycemia [2]. Over the past decade, advances in molecular engineering have led to the development of long-acting compounds and dual-incretin agonists, significantly expanding their clinical impact beyond glucose lowering to include weight reduction, cardiovascular protection, and potential anti-inflammatory and neuroprotective effects [3].

The increasing use of GLP-1RAs and related agents, particularly semaglutide and tirzepatide, has been accompanied by growing interest in their systemic and tissue-specific effects. Among these, ocular implications have become an area of active investigation. The exponential surge in prescriptions of these agents for weight management means that ophthalmologists are increasingly encountering these medications across broader, non-diabetic patient demographics, altering the traditional ocular risk profile [4]. The eye represents a metabolically sensitive organ in which microvascular, neurodegenerative, and inflammatory processes intersect [5,6]. Notably, GLP-1 receptors are expressed in multiple ocular structures, including the retina, retinal pigment epithelium, and possibly the lacrimal system, suggesting that incretin-based therapies may influence ocular physiology through both direct and indirect mechanisms [7]. Furthermore, the advent of dual GLP-1/GIP receptor agonists introduces new pharmacological complexity, although evidence regarding GIP receptor expression and function in human ocular tissues remains limited, and the ocular relevance of dual GLP-1/GIP receptor agonism should therefore be considered exploratory rather than established [8].

Clinical observations have raised important questions regarding ocular safety and potential therapeutic effects. Rapid glycemic improvement associated with potent GLP-1RAs has been linked to a phenomenon known as early worsening of diabetic retinopathy (EWDR) in susceptible patients, whereas emerging data suggest possible protective associations in conditions such as glaucoma, retinal vascular disease, and dry eye disease (DED) [9,10]. At the same time, rare but clinically relevant concerns, such as non-arteritic anterior ischemic optic neuropathy (NAION), have prompted regulatory attention and ongoing debate regarding causality and class effects [11].

Despite the rapid expansion of this field, available evidence remains heterogeneous, encompassing randomized trials, observational studies, pharmacovigilance data, and preclinical research. Differences in study design, patient populations, baseline ocular status, and comparator therapies contribute to variability in reported outcomes. Moreover, the introduction of dual-incretin agonists has added further complexity by engaging additional metabolic pathways with potentially distinct ocular implications [12,13].

In this context, a comprehensive and clinically relevant synthesis of the available evidence is warranted. This review provides an updated overview of the pharmacology of GLP-1RAs and dual-incretin therapies, with particular attention to their ocular implications. Current evidence across major ophthalmic conditions, including DR, NAION, age-related macular degeneration (AMD), glaucoma, DED and cataract, is examined alongside the biological rationale that may explain these associations. By integrating experimental findings, clinical studies, and real-world data, this review aims to clarify current knowledge, highlight areas of uncertainty, and identify priorities for future research at the intersection of metabolic medicine and eye health.

## 2. Materials and Methods

This study was conducted as a narrative review aimed at summarizing the pharmacology of GLP-1RAs and dual-incretin therapies, with particular emphasis on their ocular effects and clinical ophthalmic implications. Relevant literature was identified through a comprehensive search of PubMed, Scopus, and Web of Science from inception up to March 2026 using combinations of the following terms: “GLP-1 receptor agonist”, “semaglutide”, “liraglutide”, “dulaglutide”, “exenatide”, “lixisenatide”, “tirzepatide”, “dual-incretin agonist”, “diabetic retinopathy”, “NAION”, “optic neuropathy”, “age-related macular degeneration”, “glaucoma”, “ocular hypertension”, “dry eye disease”, “ocular surface”, “cataract”, “retinal vascular occlusion”, and “ocular complications”. Both clinical and preclinical studies were considered to provide a broad and clinically oriented overview of current evidence, including mechanistic investigations, randomized trials, observational studies, systematic reviews, and meta-analyses. Additional relevant publications were identified by manually screening the reference lists of selected articles. Studies were included if they addressed ocular outcomes and adverse events, or mechanistic pathways potentially relevant to ocular tissues in relation to GLP-1RAs or dual incretin therapies. Given the narrative design of this review and the heterogeneity of the available literature, no formal systematic quality assessment or meta-analysis was performed. Findings were therefore synthesized qualitatively, with attention to differences in study design, patient populations, therapeutic classes, and levels of evidence.

## 3. Results

### 3.1. Pharmacology and Mechanisms of Action of GLP-1 Receptor Agonists

#### 3.1.1. Physiological Basis of the GLP-1 Pathway

Glucagon-like peptide-1 (GLP-1) is an endogenous incretin hormone secreted mainly by intestinal enteroendocrine L cells in response to nutrient intake. It plays a key role in postprandial glucose homeostasis by enhancing glucose-dependent insulin secretion, suppressing inappropriate glucagon release, delaying gastric emptying, and promoting satiety through gut–brain signaling [14]. These coordinated actions reduce postprandial glycemic excursions while limiting hypoglycemia, since the insulinotropic effect of GLP-1 depends largely on ambient glucose levels [15].

Beyond pancreatic islet function, GLP-1 signaling has broader metabolic relevance because its receptors are expressed in multiple extra-pancreatic tissues, including the central nervous system, cardiovascular system, kidneys, gastrointestinal tract and eye. This widespread distribution helps explain why pharmacologic GLP-1 receptor agonism affects not only glycemic control but also body weight, appetite, cardiovascular risk, and possibly inflammatory and neuroprotective pathways. Importantly, the ability of certain GLP-1RAs to cross the blood–brain barrier facilitates direct central signaling, mediating both their potent anorexigenic effects and their neuroprotective properties across central and ocular neuronal tissues [16,17].

Native GLP-1 has a very short half-life because it is rapidly degraded by the enzyme dipeptidyl peptidase-4 (DPP-4), a limitation that drove the development of synthetic agonists with structural modifications that prolong receptor engagement [18]. Consequently, incretin-based therapies exhibit substantial pharmacologic heterogeneity, with differences in molecular structure, tissue penetrance and duration of action shaping their effects on glycemic control, weight reduction, and tolerability. In general, longer-acting GLP-1 receptor agonists, such as liraglutide and semaglutide, achieve more sustained receptor stimulation and greater reductions in HbA1c and body weight than shorter-acting compounds [19].

Taken together, these features highlight how the physiological GLP-1 pathway integrates nutrient sensing, hormonal secretion, gastric motility, and central appetite regulation into a coordinated metabolic response. This integrated biology provides the foundation for the broad therapeutic actions of GLP-1-based agents and explains why differences in receptor exposure can translate into distinct pharmacodynamic profiles and systemic outcomes.

#### 3.1.2. Structural Design and Pharmacokinetic Classification

GLP-1RAs differ markedly in molecular architecture, pharmacokinetic behavior, and dosing schedule [20]. Because native GLP-1 is rapidly degraded by DPP-4 and cleared from circulation, therapeutic compounds have been structurally modified to prolong receptor exposure and enhance biological activity. These modifications include amino acid substitutions, fatty-acid acylation, albumin binding, and other peptide engineering strategies that increase enzymatic stability and extend their half-life [21,22]. As a result, currently available agents can be broadly classified as short-acting or long-acting compounds. This distinction is clinically relevant because the pharmacokinetic profile strongly influences pharmacodynamic behavior. Short-acting agents provide intermittent receptor stimulation and predominantly reduce postprandial hyperglycemia, largely through delayed gastric emptying and meal-related insulinotropic effects [23]. By contrast, long-acting agents achieve more sustained receptor activation, resulting in greater effects on fasting glucose, overall HbA1c reduction, and body weight. These longer-acting formulations have also shown broader cardiometabolic benefits, including cardiovascular risk reduction with selected agents [24]. Structural design also helps explain why GLP-1RAs are not fully interchangeable in practice. Liraglutide and semaglutide, for example, combine prolonged receptor engagement with preserved biological potency, while semaglutide’s enhanced albumin affinity supports once-weekly administration and contributes to its strong glycemic and weight-lowering efficacy [25,26]. Weekly formulations may improve convenience and treatment adherence, although their prolonged activity can complicate the management of gastrointestinal adverse effects and peri-procedural care, particularly concerning the risk of delayed gastric emptying and pulmonary aspiration during anesthesia. Overall, the pharmacologic diversity of this drug class reflects deliberate molecular engineering that determines not only dosing frequency, but also the balance between postprandial and fasting glucose control, the magnitude of weight loss, and the breadth of extra-glycemic effects.

#### 3.1.3. Short-Acting GLP-1 Receptor Agonists: Exenatide and Lixisenatide

Exenatide twice-daily formulation and lixisenatide are the main short-acting GLP-1RAs and are characterized by relatively brief half-lives and intermittent receptor stimulation [27,28]. In contrast to longer-acting compounds—including exenatide once weekly, which represents a long-acting formulation with distinct pharmacokinetic and pharmacodynamic properties—the glucose-lowering effect of short-acting agents is driven predominantly by reduction in postprandial excursions rather than sustained control of fasting glycemia. This profile reflects a marked slowing of gastric emptying, together with meal-related enhancement of glucose-dependent insulin secretion and suppression of glucagon release [29]. As a result, these agents are particularly effective in patients whose main metabolic abnormality is postprandial hyperglycemia. Among short-acting agents, exenatide twice daily and lixisenatide once daily have shown a pronounced ability to blunt postprandial glucose peaks. This occurs because their gastric-emptying effect is preserved over time to a greater extent than with long-acting GLP-1RAs, in which tachyphylaxis may attenuate this mechanism [30]. Experimental and clinical studies with lixisenatide indicate that delayed gastric emptying is not merely an adjunctive action but a major determinant of its pharmacodynamic effect. In some settings this may reduce the postprandial insulin requirement by lowering glucose delivery to the circulation [31,32]. Because receptor exposure is less sustained, the effect of short-acting agents on fasting plasma glucose and overall HbA1c is generally more modest than that of longer-acting GLP-1RAs. Their impact on body weight is also usually smaller, and they have not demonstrated the same breadth of cardiovascular and cardiorenal benefit observed with several longer-acting molecules [33,34]. For this reason, although they remain useful in selected clinical scenarios, they are often less favored when the therapeutic priority is robust HbA1c reduction, substantial weight loss, or broader cardiometabolic risk modification. Clinically, these drugs may still be valuable when postprandial control is the dominant unmet need, including in patients whose fasting glucose is already controlled or in those receiving basal insulin. Lixisenatide, in particular, has been studied extensively in combination with basal insulin, where its prandial effect complements basal coverage [35]. However, the same pharmacologic mechanisms that drive their therapeutic efficacy also account for their tolerability profile, which is particularly relevant for short-acting agents. Because of their pronounced and sustained effect on gastric emptying, these compounds are more frequently associated with gastrointestinal adverse effects, including nausea, early satiety, and postprandial fullness, especially during treatment initiation. In addition, the marked delay in gastric emptying may influence the absorption kinetics of concomitant oral medications, a consideration that is more pronounced with short-acting than with long-acting formulations. These features are mechanistically linked to their intermittent receptor activation and preserved gastric-emptying effect, which differs from the tachyphylaxis observed with longer-acting agents. Thus, while exenatide and lixisenatide remain clinically useful in selected scenarios, their tolerability profile and pharmacodynamic characteristics should be carefully considered when individualizing therapy [36,37].

#### 3.1.4. Long-Acting GLP-1 Receptor Agonists: Liraglutide, Dulaglutide, and Semaglutide

Long-acting GLP-1RAs, including liraglutide, dulaglutide, and semaglutide, are characterized by sustained receptor activation throughout the dosing interval, resulting in more stable and consistent glycemic control compared with short-acting compounds. This prolonged activity is achieved through structural modifications that delay degradation and clearance, allowing sustained GLP-1 receptors exposure and more pronounced effects on fasting plasma glucose and overall HbA1c [38]. In contrast to short-acting agents, the impact of gastric emptying becomes less dominant over time, presumably due to tachyphylaxis, while central and pancreatic mechanisms, such as appetite suppression and glucose-dependent insulin secretion, play a larger role in their therapeutic profile [39]. Beyond glycemic control, long-acting GLP-1RAs have demonstrated clinically relevant benefits on body weight and cardiometabolic outcomes. Liraglutide was among the first agents in this class to show reductions in major adverse cardiovascular events, establishing a paradigm shift in the management of patients with T2DM and high cardiovascular risk [40]. Subsequent cardiovascular outcome trials have confirmed cardiovascular benefit for multiple long-acting GLP-1 receptor agonists, although this effect is not consistent across the entire class, supporting their integration into contemporary treatment guidelines not only for glucose lowering but also for cardiovascular risk reduction [38]. Pharmacokinetic differences within this group influence both dosing frequency and clinical performance. Liraglutide, administered once daily, provides sustained but shorter receptor exposure compared with once-weekly agents such as dulaglutide and semaglutide, which achieve prolonged systemic levels through albumin binding or large molecular constructs that reduce renal clearance. These weekly formulations improve treatment convenience and adherence while maintaining steady pharmacodynamic effects over time [41]. Among the available agents, semaglutide has shown particularly strong efficacy in both glycemic control and weight reduction. Its long half-life and high receptor potency translate into substantial reductions in HbA1c and consistent weight loss across clinical trials, including in non-diabetic populations [42,43]. Comparative analyses and meta-analyses generally indicate greater weight loss with semaglutide than with liraglutide or dulaglutide, even when glycemic outcomes are similar. This enhanced efficacy is likely related to both its pharmacokinetic properties and its deeper central effects on appetite regulation [44,45]. Overall, long-acting GLP-1RAs represent a cornerstone of modern incretin-based therapy. Their ability to provide sustained metabolic control, promote weight loss, and reduce cardiovascular risk distinguishes them from earlier short-acting agents and underscores the importance of pharmacologic design in shaping clinical outcomes. These properties have also paved the way for the development of newer incretin-based strategies, including multi-receptor agonists, which aim to further enhance metabolic efficacy through complementary mechanisms of action.

#### 3.1.5. Dual-Incretin Agonism: Tirzepatide and the GLP-1/GIP Axis

Tirzepatide has expanded incretin pharmacology by combining GLP-1 receptor agonism with activation of the glucose-dependent insulinotropic polypeptide (GIP) receptor. This dual mechanism appears to enhance metabolic efficacy beyond that typically achieved with single-receptor GLP-1 agonists, producing greater reductions in HbA1c and body weight across clinical studies. In addition to potentiating glucose-dependent insulin secretion and suppressing glucagon, tirzepatide improves satiety and reduces energy intake, thereby contributing to its pronounced weight-lowering effect [46,47]. Clinical reviews and meta-analyses consistently describe tirzepatide as one of the most potent currently available incretin-based therapies for T2DM and obesity [48,49,50]. Its pharmacologic profile is not simply the sum of two parallel receptor effects. Pharmacological signaling studies indicate that tirzepatide closely mimics the actions of native GIP at the GIP receptor, while acting as a biased agonist at the GLP-1 receptor. In this context, tirzepatide preferentially promotes cAMP generation over β-arrestin recruitment, a signaling pattern associated with reduced GLP-1 receptor internalization compared with native GLP-1. This biased agonism may attenuate receptor desensitization and contribute to its sustained pharmacodynamic activity [51]. This integrated GLP-1/GIP signaling may also influence adipose tissue biology, lipid metabolism, and central appetite pathways, helping explain the magnitude of its effects on weight and cardiometabolic risk markers [52]. Compared with traditional GLP-1RAs, tirzepatide has shown superior weight reduction in multiple comparative analyses, while maintaining a safety profile broadly consistent with the incretin class, particularly with respect to gastrointestinal adverse events. These features have positioned tirzepatide as a major therapeutic advance not only in diabetes care but also in obesity and broader metabolic disease [53]. At the same time, its expanding use has raised new questions regarding tissue-specific effects, long-term safety, and potential benefits beyond glycemic control, including cardiovascular, hepatic, and neuroendocrine outcomes [54]. Overall, tirzepatide illustrates how dual-incretin receptor engagement can translate into clinically meaningful gains in efficacy. Its emergence marks a shift from conventional GLP-1 receptor agonism toward multi-receptor incretin therapeutics designed to amplify metabolic benefit while preserving the glucose-dependent safety advantages of the incretin pathway. Importantly, it is precisely this rapid and profound metabolic shift—particularly the steep decline in HbA1c observed with potent incretin-based therapies such as semaglutide and tirzepatide—that has brought their potential ocular implications into sharp clinical focus. However, whether and how this enhanced metabolic efficacy translates into modification of ocular risk profiles remains a critical area of ongoing investigation. In this context, recent reviews have also highlighted the emerging but still incompletely defined role of GLP-1 receptor signaling in ocular tissues, supporting the need for further mechanistic and clinical investigation in this field [13].

#### 3.1.6. Adverse Effects, Tolerability, and Practical Pharmacologic Considerations

The adverse-effect profile of incretin-based therapies, particularly GLP-1RAs, is primarily driven by their gastrointestinal mechanisms of action. These agents, widely used as injectable treatments for T2DM and increasingly for obesity, improve glycemic control and several cardiometabolic parameters without inducing hypoglycemia when used in combination with metformin or thiazolidinediones. The most common adverse effects include nausea, vomiting, early satiety, abdominal discomfort, and delayed gastric emptying, which are typically dose-dependent and occur most frequently during treatment initiation and dose escalation. Although these symptoms often attenuate over time, they remain a key determinant of treatment adherence and persistence [55].

Tolerability varies across agents and formulations: short-acting compounds tend to exert more pronounced, meal-related effects on gastric emptying, whereas long-acting and once-weekly formulations improve convenience but may prolong gastrointestinal symptoms in susceptible individuals. These pharmacokinetic differences are clinically relevant in specific settings, such as peri-procedural management, where delayed gastric emptying may increase the risk of pulmonary aspiration [56].

Additional safety considerations include rare but potentially serious events, such as pancreatitis and acute kidney injury, with the latter often secondary to dehydration from gastrointestinal losses. Early concerns regarding pancreatic and thyroid malignancies, suggested by preclinical and pharmacovigilance data, have not been consistently confirmed by large meta-analyses [57,58].

Overall, current evidence supports a favorable cardiovascular safety profile for GLP-1RAs, although long-term safety across diverse populations, including non-diabetic individuals using these agents for weight loss, remains under active investigation [55]. In clinical practice, treatment selection should be individualized based on efficacy goals, tolerability, comorbidities, and dosing preferences. Beyond these established systemic effects, increasing attention has been directed toward the potential ocular implications of incretin-based therapies, which are explored in the following sections.

### 3.2. Diabetic Retinopathy

Diabetic retinopathy (DR) is one of the most common microvascular complications of diabetes and remains a major cause of vision loss worldwide. Its pathophysiology involves neurovascular dysfunction, inflammation, oxidative stress, and progressive retinal neurodegeneration in parallel with vascular injury [59,60]. Since DR can transiently worsen during periods of rapid glycemic improvement, ocular safety is a clinically relevant consideration when initiating potent glucose-lowering therapies such as GLP-1RAs [61,62].

The main concern stems from the SUSTAIN-6 trial, where semaglutide was associated with more DR complications than placebo [63]. However, this finding has generally been interpreted within the framework of early worsening rather than direct retinal toxicity. Subsequent analyses reported that the excess risk was concentrated largely in participants with pre-existing retinopathy, concomitant insulin use, and marked early HbA1c reduction, supporting the view that baseline retinal status and the magnitude of glucose lowering are central determinants of risk [64,65]. A population-based real-world study further suggested that rapid HbA1c decline does not uniformly translate into early worsening, particularly in milder baseline retinopathy, reinforcing the likelihood that susceptibility differs across patient subgroups [66].

Meta-regression across cardiovascular outcome trials suggests that diabetic retinopathy outcomes may correlate more closely with the magnitude of early HbA1c reduction than with exposure to any specific GLP-1 receptor agonist [67]. However, these analyses support an association rather than a direct causal relationship, and should therefore be interpreted with caution. Similarly, other meta-analyses have reported either a small increase in early-stage DR with some comparator structures or no overall increase when semaglutide trials are pooled more broadly, again suggesting that study design, comparator choice, and baseline risk strongly influence the observed effect [68,69].

Real-world evidence has also been mixed. Earlier large cohort studies did not identify a consistent increase in retinopathy complications among GLP-1RA users [70,71]. A recent study reported that GLP-1RAs were associated with increased DR diagnoses but fewer sight-threatening complications [9]. This apparently divergent finding may reflect increased ascertainment, differential ophthalmic surveillance, or diagnostic coding, rather than a uniform increase in clinically severe diabetic retinopathy. Other large cohort studies did not find heightened risk of advance DR with GLP-1RAs [72,73]. Furthermore, other retrospective studies have reported a significantly lower risk of DR for patients treated with GLP-1RAs [74,75].

Also, the evidence on tirzepatide is mixed. A real-world cohort study showed findings consistent with early worsening, particularly in patients with more advanced baseline retinopathy phenotypes [76]; whereas other recent cohort analyses reported lower short-term risks of incidence or worsening DR outcomes and fewer retina-related complications relative to comparator cohorts [77,78]. Taken together, these findings again suggest that baseline retinal status, the glycemic trajectory, and comparator selection are likely critical modifiers of observed risk.

Systematic syntheses published in late 2025 and early 2026 are broadly consistent with this interpretation. Overall, a robust average increase in DR development or progression across the GLP-1RA class is not reported; however, substantial heterogeneity between randomized and observational evidence, between individual agents, and across populations with differing baseline retinal risk is reported [79,80].

Taken together, current evidence does not support routinely avoiding GLP-1RAs in patients with DR. The main safety concern remains the possibility of transient early worsening, especially in patients with pre-existing retinopathy who experience a large and rapid fall in HbA1c. Outside that context, more recent randomized syntheses and contemporary real-world studies have not shown a consistent excess of sight-threatening DR events across the class. From a practical standpoint, the most defensible approach is not drug avoidance, but attention to baseline retinal status through screening before treatment initiation in higher-risk patients and closer ophthalmic follow-up after initiation or dose escalation [62,81,82].

Moreover, comparative evidence on sodium-glucose cotransporter-2 inhibitors (SGLT2i) has generally been neutral to favorable for DR outcomes in real-world settings, with some studies suggesting lower risks of incident or progressive retinopathy than comparator therapies, including in head-to-head analyses against GLP-1RAs. These data may be relevant when both drug classes are appropriate, particularly in patients with established retinopathy, although they do not alter the central conclusion that GLP-1RAs-associated ocular risk appears to be driven more by patient context and glycemic dynamics than by consistent direct retinal toxicity [83,84,85].

A drug-specific overview of DR risk across major incretin-based therapies is provided in Table 1.

### 3.3. Non-Arteritic Anterior Ischemic Optic Neuropathy

NAION is an acute ischemic optic neuropathy characterized by sudden, usually painless, unilateral vision loss in middle-aged and older adults. It is generally considered an ischemic event at the level of the optic nerve head, although its pathogenesis remains incompletely understood and diagnostic classification may be challenging, particularly in patients with atypical features or overlapping anterior ischemic optic neuropathy phenotypes [91]. Interpretation of possible drug-safety signals is therefore especially complex in populations with T2DM or obesity, who already carry a substantial baseline burden of vascular risk factors. In this context, NAION has emerged as one of the most closely scrutinized neuro-ophthalmic safety concerns in relation to GLP-1RAs use.

Attention to this issue increased markedly after the report by Hathaway et al., which described a higher cumulative incidence of NAION among semaglutide-exposed patients in a neuro-ophthalmology registry, with a hazard ratio (HR) of 4.28 in patients with T2DM and 7.64 in overweight or obese individuals [11]. Since that analysis was based on a single-center specialty registry, it was widely interpreted as hypothesis-generating rather than causal evidence. This cautious interpretation was echoed by the American Academy of Ophthalmology (AAO) [92]. Regulatory agencies subsequently adopted a more formal position: on 6 June 2025, the EMA’s Pharmacovigilance Risk Assessment Committee concluded that NAION should be considered a very rare adverse reaction to semaglutide-containing medicines [93]; on 27 June 2025, the WHO issued a medical product alert [94]; and in February 2026, the MHRA advised urgent ophthalmologic assessment for sudden visual loss during semaglutide treatment and discontinuation if NAION is confirmed [95].

Semaglutide-specific observational evidence has been mixed, but, overall, suggests that any increase in NAION risk, if present, is likely small in absolute terms and more modest than initially feared. In a Danish-Norwegian cohort of 424,152 individuals with T2DM, once-weekly semaglutide was associated with an approximately twofold increase in 5-year NAION risk [96]. Another multicenter study later reported an HR ranging from 1.44 to 2.27 depending on the comparator and analytic approach, concluding that the association was smaller than originally reported and not consistently elevated across all analyses [97]. Similarly, Hsu et al. found no excess risk in the first year of semaglutide exposure, but higher hazards emerged after longer follow-up in diabetes cohorts [98]. In a target-trial emulation among US veterans with T2DM, semaglutide initiators had a higher risk of incident NAION than SGLT2i initiators, although the absolute incidence remained low [99]. A UK cohort study comparing GLP-1RAs to DPP-4 inhibitors similarly found an increased 1-year NAION risk, with higher risk during the first 6 months of use and in patients with ≥1% HbA1c reduction, younger age, male sex, or smoking history [100]. By contrast, not all large observational datasets have confirmed an increased risk. A multinational population-based study found no significant association between semaglutide and NAION in cohorts with T2DM, obesity, or both [101]. Likewise, another US cohort study found no increased risk of NAION or ischemic optic neuropathy with semaglutide or GLP-1RAs overall [102].

Case reports and pharmacovigilance analyses have contributed to concern regarding a possible association between incretin-based therapy and NAION, but these data require cautious interpretation. Semaglutide has featured prominently in several reports and spontaneous adverse-event analyses, including cases occurring in patients with crowded optic discs, small Bruch’s membrane opening diameters, or other anatomical susceptibility factors [103,104,105,106]. Importantly, these structural features are themselves established or plausible risk factors for NAION and should be interpreted as potential confounders or susceptibility markers rather than evidence of drug-induced pathology. Therefore, such reports primarily help identify phenotypes that may warrant closer clinical attention, but they cannot establish a mechanistic or causal relationship between GLP-1RA exposure and NAION. In addition, the apparent predominance of semaglutide should not be interpreted as evidence of greater intrinsic biological toxicity compared with other GLP-1RAs. Semaglutide has experienced unprecedented prescription growth, is widely used across both diabetes and obesity populations, and has been subject to substantial public, scientific, and regulatory attention. These factors may increase the likelihood of stimulated reporting, notoriety bias, and differential ophthalmic surveillance. Moreover, pharmacovigilance databases lack reliable exposure denominators, standardized diagnostic adjudication, and complete adjustment for vascular, metabolic, and anatomical risk factors. Therefore, although spontaneous reports are useful for signal detection, they cannot establish incidence, causality, comparative drug-specific risk, or drug-induced optic nerve pathology. The broader question of a GLP-1RAs class effect remains less clear. A 2026 meta-analysis reported an association between GLP-1RA exposure and NAION, but the absolute risk was very low, with an estimated absolute NAION risk of 0.09% and a risk difference of 0.037% [107]. Importantly, randomized evidence provides a relevant counterbalance to observational safety signals: a meta-analysis of randomized controlled trials found no significant increase in optic nerve or other vision-threatening adverse events with GLP-1RAs overall, including no significant increase in ischemic optic neuropathy [108]. This RCT-based evidence is less susceptible to confounding by indication, differential ophthalmic surveillance, and imbalance in baseline vascular risk than retrospective database studies. However, it should also be interpreted cautiously, because most trials were not primarily designed to detect NAION, ophthalmic events were rare, and standardized neuro-ophthalmic adjudication was not consistently available. A systematic review with GRADE assessment reported a pooled adjusted HR of 1.63 for semaglutide versus non-GLP-1RA comparators, but emphasized substantial heterogeneity, fragility, and low to very low certainty of evidence; notably, the association appeared stronger in T2DM populations than in obesity/overweight populations [109]. This is clinically relevant because most positive associations have arisen from retrospective diabetes cohorts, a population already enriched for vascular and microvascular comorbidities that independently increase susceptibility to optic nerve ischemia [110]. Baseline diabetes severity, insulin use, hypertension, dyslipidemia, obstructive sleep apnea, smoking, rapid glycemic improvement, and incomplete characterization of optic disc anatomy may all confound observed associations. Semaglutide-focused reviews and meta-analyses have similarly emphasized that the available evidence is driven largely by observational studies in diabetes populations, whereas randomized trials and weight-management cohorts provide less consistent support for increased risk [111,112,113]. Taken together, current evidence does not support a simple uniform class effect. Although the signal has been most frequently discussed in relation to semaglutide, this may reflect differences in exposure, indication, surveillance intensity, and reporting behavior rather than a true difference in biological toxicity. Any potential risk appears uncommon in absolute terms and remains incompletely characterized. Overall, the relationship between GLP-1RAs and NAION should be interpreted cautiously and according to the strength of the available evidence. Randomized trial data have not demonstrated a significant increase in optic nerve or major vision-threatening adverse events, whereas observational and pharmacovigilance studies have generated a potential semaglutide-associated signal, mainly in T2DM cohorts, that remains rare in absolute terms and requires further investigation. The resulting picture is therefore not one of a broad or frequent ocular toxicity, but rather of a rare event for which relative risk may be increased in selected settings while absolute risk remains low. Importantly, T2DM itself is a major vascular risk state, and many exposed cohorts include patients with long-standing diabetes, insulin use, hypertension, dyslipidemia, sleep apnea, or other comorbidities that may independently predispose to optic nerve ischemia. Case reports should be weighted accordingly: they may highlight susceptible anatomical phenotypes, such as crowded optic discs, but they should not be interpreted as proof of drug-induced optic nerve ischemia. In practical terms, current evidence supports informing patients receiving semaglutide to seek urgent assessment for sudden painless visual loss, in line with current regulatory guidance. Further prospective studies with standardized NAION adjudication, careful comparator selection, and more complete characterization of optic disc anatomy and vascular risk are needed to clarify whether the observed association reflects a semaglutide-specific pharmacologic effect, confounding by indication, or a combination of these factors. A comprehensive overview of the available evidence on NAION risk associated with GLP-1RAs, including observational studies, pharmacovigilance data, and meta-analyses, is summarized in Table 2.

### 3.4. Age-Related Macular Degeneration

AMD is a chronic degenerative disorder of the macula and a leading cause of irreversible central vision loss in older adults. It is classified into two forms: non-exudative (dry or non-neovascular) AMD and exudative (wet or neovascular) AMD. Despite major advances in anti-VEGF therapy for neovascular AMD, effective preventive or disease-modifying strategies remain limited, particularly for the dry form [114].

Clinical evidence on the association between GLP-1RAs and AMD is currently derived almost entirely from retrospective electronic health record (EHR) and administrative-claims analyses, predominantly from North American datasets. As a result, the available evidence remains heterogeneous and should be interpreted within the limitations inherent to real-world observational research, including outcome misclassification, incomplete phenotyping, residual confounding, and variable ophthalmic surveillance intensity [114,115].

In a large TriNetX-based retrospective cohort study evaluating chronic ocular outcomes in older adults, GLP-1RA exposure was associated with a reduced hazard of non-exudative AMD compared with metformin, insulin, and statins, with the difference becoming most apparent after approximately 3 years of follow-up. In the same study, GLP-1RAs use was also associated with a lower risk of exudative AMD compared with insulin at 3 years [116]. A subsequent AMD-focused analysis from the same research group based on the TriNetX network found that GLP-1RAs prescription was associated with a lower risk of non-neovascular AMD at 1, 2, and 3 years compared with alternative glucose-lowering therapies. GLP-1RA use was also associated with a lower risk of neovascular AMD across all time points relative to both glucose-lowering and lipid-lowering comparator groups, and no significant effect was observed on conversion from baseline non-neovascular to neovascular AMD [117]. Reinforcing this association, McLaughlin et al., in a matched cohort of 600,816 adults with T2DM and no prior AMD, reported a lower incidence of both non-exudative AMD and exudative AMD among GLP-1RA users. Among patients with early or intermediate non-exudative AMD at baseline, exposure to GLP-1RAs was additionally associated with a 29% and 27% relative risk reduction in progression to advanced non-exudative AMD and to exudative AMD, respectively. These protective signals biologically align with the known anti-inflammatory and anti-oxidative properties of GLP-1RAs, which may mitigate the chronic RPE stress central to early AMD pathogenesis [118].

In contrast, the strongest finding suggesting possible harm derived from GLP1-RAs use comes from a large population-based matched cohort study from Ontario, Canada. In this analysis, GLP-1RA exposure was associated with a 2-fold higher risk of incident neovascular AMD in older adults with diabetes, with a reported duration-dependent pattern suggesting that longer exposure durations corresponded to a higher risk [119]. This study represents the principal counterpoint to the apparently protective findings observed in US-based EHR analyses and has prompted debate regarding whether GLP-1RAs could exert divergent effects depending on disease stage, retinal susceptibility, or patient characteristics [115].

These apparently discordant findings likely reflect important methodological differences across studies. Analyses suggesting reduced AMD risk have generally relied on active-comparator designs, contrasting GLP-1RAs with other glucose- or lipid-lowering therapies, whereas the Ontario study compared exposed patients with unexposed individuals with diabetes [116,117,119]. Outcome definitions also differed substantially, with some studies evaluating incident non-neovascular AMD, others incident neovascular AMD, and others progression or conversion endpoints [116,117,119]. In addition, key determinants of AMD risk, such as smoking status, baseline retinal phenotype, disease stage, and frequency of ophthalmic follow-up, are incompletely captured in claims and EHR datasets and may contribute to residual confounding or surveillance bias [115].

In summary, current evidence suggests that the relationship between GLP-1RAs and AMD is unlikely to represent a uniform class effect. The protective associations reported in TriNetX-based studies may reflect systemic metabolic benefits, whereas the increased risk of incident neovascular AMD observed in the Ontario cohort may be linked to proangiogenic mechanisms or to the effects of rapid glucose normalization [120]. However, these interpretations remain hypothetical and should not be considered evidence of a true biologically stage-dependent effect. The apparently discordant findings may instead reflect important methodological differences between studies, including comparator selection, residual confounding, heterogeneity in AMD definitions and staging, and differences in ophthalmic surveillance intensity. Accordingly, whether GLP-1RAs exert differential effects across AMD stages remains unresolved. Further prospective studies with standardized AMD staging and detailed metabolic characterization are needed to clarify causality and identify susceptible subgroups.

The available evidence on AMD is summarized in Table 3.

### 3.5. Ocular Hypertension and Glaucoma

Beyond intraocular pressure (IOP), neuroinflammatory signaling, glial reactivity, and retinal ganglion cell stress play a significant role in glaucomatous neurodegeneration. Specifically, the activation of GLP-1 receptors on retinal microglia and macroglia has been proposed to shift these cells away from a neurotoxic, pro-inflammatory state toward a neuroprotective phenotype, offering a biological rational for potential disease-modifying effects in glaucoma [122].

Preclinical data provide some mechanistic support, but not definitive evidence of neuroprotection. In a rat model of ocular hypertension (OHT), systemic semaglutide delayed IOP elevation and attenuated changes consistent with reactive astrocytosis and vascular remodeling. However, it did not improve retinal ganglion cell survival [123].

Across clinical observational studies, a lower incidence of primary open-angle glaucoma (POAG) among patients treated with GLP-1RAs was recorded. In a large international EHR-based analysis, Muayad et al. compared GLP-1RAs with metformin in patients with T2DM and found lower risks of POAG at 1, 2, and 3 years, with corresponding relative risks of 0.59, 0.50, and 0.59, respectively. The same study also reported lower risks of OHT across the same time points, as well as reduced initiation of first-line glaucoma treatment, including topical therapy and laser trabeculoplasty [124].

Similarly, in a broader comparative effectiveness study of chronic age-related ocular disease, Allan et al. found that GLP-1RA exposure was associated with a lower risk of POAG, most clearly in comparison with insulin after 3 years, whereas no consistent long-term association was observed for OHT across medication comparators [116].

More recently, head-to-head comparisons have extended beyond the use of selective GLP-1RAs. Hong et al. reported lower risks of POAG, OHT, and the need for glaucoma treatment in patients receiving tirzepatide compared with selective GLP-1RAs [125].

A similar pattern was observed also in non-diabetic populations. In a TriNetX study of patients with overweight or obesity but without diabetes, Vasu et al. reported lower risks of both POAG and OHT at 3 and 5 years among GLP-1RA users compared with patients receiving alternative weight-loss medications including orlistat, phentermine-topiramate, bupropion-naltrexone, or setmelanotide [126].

Several limitations recur throughout the evidence reported. Most studies rely on retrospective EHR or claim data, with variable ascertainment of POAG and OHT and limited capture of clinically meaningful ophthalmic parameters such as true IOP trajectories, baseline glaucoma stage, optic nerve appearance, OCT-derived RNFL or GCC measures, visual field progression, and treatment adherence. Residual confounding also remains likely, particularly because GLP-1RAs are often prescribed in patient groups that differ systematically from comparator cohorts in terms of metabolic profile, cardiovascular risk, health-seeking behavior, and frequency of specialist follow-up [127,128]. A summary of the main studies addressing glaucoma and ocular hypertension in relation to GLP-1RAs use is provided in Table 4.

Overall, current evidence supports an association between GLP-1RA exposure and a lower recorded incidence of POAG, whereas the signal for OHT is less reproducible across datasets. Prospective studies with standardized protocols will be necessary to determine whether GLP-1RAs confer genuine ophthalmic benefit and whether any such effect is class-wide or agent-specific.

### 3.6. Dry Eye Disease

Dry eye disease (DED) represents a frequent ocular surface disorder often associated with T2DM and obesity, conditions characterized by systemic low-grade inflammation and chronic metabolic dysfunction. Previous studies have demonstrated an increased prevalence of DED among patients with T2DM, with an occurrence rate from 17% to over 50%, relying on specific population criteria, diagnostic characteristics, and disease history [129]. Moreover, accumulated evidence considers obesity as a risk factor for DED, predominantly through evaporative mechanisms allied with meibomian gland disease, lipid profile abnormality, and persistent inflammation [69]. Within this frame of reference, the improvement in body weight, glycemic control, and systemic inflammatory status observed with GLP-1RAs therapy has generated interest in the possibility of indirect beneficial effects on the ocular surface, although current evidence remains limited [130]. In addition, in patients with T2DM, improved metabolic control has been associated with a partial recovery of corneal nerve function and an attenuation of ocular surface inflammation [131].

In line with this hypothesis, preliminary clinical observations have suggested that GLP-1RAs use may be associated with improvements in selected tear film parameters and tear production, potentially mediated through anti-inflammatory effects on the lacrimal system [132]. In a case–control study involving patients with T2DM, dry eye parameters were analyzed in individuals treated with GLP-1RAs compared with controls not receiving GLP-1–based therapy. The authors reported improved tear production and tear film stability in the treated group, although the observational design and limited available evidence preclude definitive conclusions regarding causality or clinical efficacy [132]. The authors hypothesized that these findings may be related to the expression of GLP-1 receptors in lacrimal gland tissue, as well as to the known anti-inflammatory and vasoprotective effects of the GLP-1 signaling pathway. At present, however, these mechanisms remain largely speculative in humans and are supported primarily by preclinical evidence. Supporting evidence for this mechanism has been demonstrated in murine studies, where GLP-1RAs eye drops were shown to suppress lacrimal gland inflammation in a mouse model of type 1 diabetes [133]. Furthermore, Su et al. compared the effect of SGLT2i with GLP-1Ras, showing that patients with T2DM, first initiated on SGLT2i, might encounter a decreased prevalence of DED (9 events/1000 person-years) compared to those receiving GLP-1RAs (11.5 events/1000 person-years) [10]. Also, another Taiwanese retrospective study analyzed T2DM patients dividing them into those receiving GLP-1RAs treatment and those without therapy. The treated group showed a significantly lower percentage of subsequent DED and superficial keratitis compared to the control group. In subgroup analyses, the association between GLP-1RAs use and DED was more pronounced in patients <60 years [134].

Nevertheless, despite the reported benefits, the systemic side-effect of GLP-1RAs, especially gastrointestinal symptoms associated with reduced oral intake, may predispose certain patients to dehydration, which could exacerbate DED symptoms and partially offset ocular surface improvement [135]. In addition, the absence of prospective studies evaluating the prevalence and severity of DED before and during GLP-1RAs therapy remains a current gap. Overall, available evidence remains preliminary and largely observational, and further prospective studies incorporating standardized ocular surface assessments are required before any definitive conclusions regarding the role of GLP-1RAs in DED can be established. Increased awareness among both eye care specialists and GLP-1RAs prescribers is warranted to optimize systemic metabolic benefits while minimizing potential adverse ocular surface outcomes.

### 3.7. Cataract

The relationship between GLP-1RAs therapy and cataract development has so far primarily been evaluated through large retrospective observational studies [121,136].

In a real-world cohort study of T2DM adults older than 40 years, cataract incidence was compared among users of sodium–glucose cotransporter-2 inhibitors (SGLT2i), GLP-1RAs, and dipeptidyl peptidase-4 inhibitors (DPP-4i). After propensity score matching, SGLT2i use was associated with a lower risk of cataract formation compared with GLP-1RAs and DPP-4i. Importantly, these results do not indicate an increased cataract risk associated with GLP-1RAs therapy but rather suggest a relative protective association specific to SGLT2 inhibition within the diabetic population [136].

In another cohort study examining overweight and obese non-diabetic adults older than 55 years, GLP-1RAs drugs (semaglutide and liraglutide) were associated with a significantly reduced long-term risk (at 5, 7 and 10 years) of age-related cataract development compared with either other pharmacologic weight-loss therapies (lorcaserin, setmelanotide, diethylpropion, sibutramine, fenfluramine, mazindol, phentermine, or orlistat) or no weight-loss treatment [121].

Both studies are limited by their retrospective design and reliance on administrative databases, which introduce potential residual confounding related to metabolic control, comorbidity burden, and healthcare utilization. Taken together, these findings suggest that GLP-1RAs are not associated with an increased risk of cataract formation and instead may confer a protective association in certain non-diabetic populations. Nonetheless, a direct causal relationship between GLP-1RAs therapy and cataract risk cannot be established and caution should be exercised when interpreting these data.

### 3.8. Retinal Vascular Changes

Evidence regarding retinal vascular effects of GLP-1RAs varies by drug class, and careful attention to study scope is required.

A recent multinational retrospective cohort study specifically addressed the comparative risk of retinal vein occlusion (RVO) in patients with T2DM newly prescribed GLP-1RAs versus DPP-4i. Among 79, 486 propensity score-matched participants drawn from real-world electronic health record data, GLP-1RAs use was associated with a significantly lower risk of incident RVO (HR 0.73; 95% CI 0.54–0.98) and branch retinal vein occlusion (BRVO) (HR 0.62; 95% CI 0.41–0.95) over 5 years compared with DPP-4i use. This protective association was particularly evident across high-risk subgroups, including patients older than 50 years, those with baseline HbA1c ≥ 8%, BMI ≥ 30 kg/m^2^, or diabetes duration ≥ 3 years [137]. These findings suggest that GLP-1RAs may confer a lower risk of specific retinal vascular occlusive events relative to DPP-4i, possibly reflecting differential effects on systemic vascular risk factors or microvascular integrity.

Since these results derive from a retrospective cohort with inherent limitations, prospective randomized studies are necessary to confirm potential class-specific retinal vascular effects of glucose-lowering agents.

## 4. Discussion

The increasing use of GLP-1RAs and dual-incretin therapies has broadened the intersection between metabolic medicine and ophthalmology, bringing renewed attention to their potential ocular effects. These agents, originally developed for glycemic control, are now recognized as systemic modulators with complex actions that extend to vascular, inflammatory, and neurodegenerative pathways relevant to the eye [7]. The available evidence suggests that their ocular impact is multifaceted and highly context-dependent, influenced by baseline disease status, the speed of metabolic improvement, and individual susceptibility factors.

Preclinical and translational studies suggest that GLP-1 receptor signaling may exert potentially beneficial effects through the reduction in oxidative stress, modulation of inflammatory pathways, stabilization of the blood–retinal barrier, and neuroprotection of retinal ganglion cells [138]. These processes may contribute to a more favorable retinal microenvironment and could partially explain the signals of protection reported in observational studies of DR, glaucoma, and ocular surface disease. However, these findings must be interpreted cautiously, as causal inference remains limited, and evidence from randomized trials is still evolving. In parallel, clinical data suggest that rapid glycemic improvement, particularly with potent agents such as semaglutide and tirzepatide, may transiently destabilize retinal homeostasis, especially in patients with pre-existing microvascular disease [67].

In clinical practice, these considerations support a risk-stratified and individualized approach rather than a universal strategy. Indeed, routine enhanced ophthalmic assessment should be prioritized in individuals at higher ocular risk, including those with diabetes, pre-existing DR, poor baseline glycemic control or anticipated rapid HbA1c reduction, prior NAION, crowded optic disc anatomy, or relevant vascular comorbidities. In such patients, documenting baseline ophthalmic status and ensuring recent comprehensive eye examination is advisable. Where appropriate, baseline evaluation with dilated fundus examination, optic nerve assessment, and adjunctive imaging (e.g., OCT) may provide useful reference points for follow-up. Patient education is equally critical. Individuals should be informed about symptoms that warrant prompt ophthalmologic evaluation, including sudden painless vision loss suggestive of optic neuropathy, new visual field defects, increased floaters or flashes potentially indicating retinal pathology, and distortion of central vision that may reflect macular involvement. Decisions regarding alternative glucose-lowering therapies in patients at potential risk of NAION should be individualized, taking into account overall metabolic benefit, ocular risk profile, and patient preferences, rather than applied as uniform recommendations. Shared decision-making is therefore essential in balancing systemic benefits against uncertain ocular risks.

Effective management also requires interdisciplinary communication. Ophthalmologists should routinely inquire about the use of GLP-1RAs and related therapies, while endocrinologists and primary care physicians should document ocular history and ensure appropriate referral when indicated. Coordinated care is particularly important for patients at higher risk, in whom closer monitoring and tailored therapeutic strategies may help mitigate potential complications while preserving systemic benefits.

Interpretation of the current literature is complicated by important methodological limitations. Much of the available evidence derives from retrospective cohort studies and pharmacovigilance databases, which are inherently susceptible to confounding, selection bias, and incomplete outcome ascertainment. This limitation is particularly relevant for NAION, because most positive associations have been reported in retrospective T2DM cohorts already enriched for vascular comorbidity and microvascular disease, making it difficult to separate a potential drug-related signal from baseline susceptibility, glycemic shifts, and confounding by indication. Factors such as the “healthy user effect,” differences in baseline disease severity, and variability in ophthalmologic follow-up may influence the reported associations. Although some studies have attempted to address these issues through advanced statistical approaches, current evidence should be interpreted as associative rather than definitive, and residual confounding cannot be excluded [79,128].

Looking ahead, ongoing and future studies will be critical in refining our understanding of these relationships. Trials such as the FOCUS study, evaluating the progression of DR in patients treated with semaglutide, are expected to provide more definitive evidence in the coming years [139]. Beyond individual trials, there is a clear need for prospective ophthalmic surveillance studies in patients initiating GLP-1RAs or dual-incretin agonists, ideally incorporating standardized baseline and follow-up assessments of the retina and optic nerve. Such studies should include predefined ophthalmic endpoints, systematic capture of visual symptoms, optic nerve imaging, retinal imaging, glycemic trajectory, vascular comorbidities, and absolute-risk reporting. For NAION specifically, mechanistic investigations are needed to clarify whether incretin-based therapies may influence optic nerve head perfusion, endothelial function, nocturnal hypotension, vascular autoregulation, or susceptibility to ischemia in anatomically predisposed eyes. Subgroup analyses should also focus on individuals with crowded optic discs, reduced Bruch’s membrane opening diameter, prior NAION, long-standing T2DM, insulin use, obstructive sleep apnea, or other vascular risk factors. Additional priorities include agent-specific and dose–response analyses, as well as the identification of biomarkers that may predict susceptibility to adverse or beneficial ocular effects. Emerging research directions are also exploring the possibility of directly targeting ocular tissues through incretin-based mechanisms. Experimental studies on topical GLP-1RAs suggest that localized delivery could harness neuroprotective and anti-inflammatory effects while minimizing systemic exposure, opening new therapeutic avenues for retinal and optic nerve diseases [140]. Such approaches remain in early stages but highlight the broader translational potential of incretin biology in ophthalmology.

In summary, incretin-based therapies represent a rapidly evolving field with important implications for eye health. While their metabolic benefits are well established, their ocular effects remain incompletely defined. Clinical recommendations should therefore remain proportionate to the current level of evidence, emphasizing individualized risk assessment, appropriate baseline evaluation in selected patients, and interdisciplinary collaboration. As evidence continues to evolve, integrating mechanistic, observational, and clinical trial data will be essential to define the role of these therapies at the interface between systemic disease and ophthalmology.

## Figures and Tables

**Table 1:** Drug-specific DR risk with incretin-based therapies.

Agent	First Author	DR Risk	Key Evidence	Practical Clinical Implication
Semaglutide	Aroda et al. (2023) [63]; Vilsbøll et al. (2018) [64]; Marso et al. (2016) [65]; Bethel et al. (2021) [67]	Possible early worsening in high-risk patients; no consistent signal for increased sight-threatening DR across contemporary real-world studies.	SUSTAIN-6 showed an excess of DR complications compared to placebo; post hoc analyses link this signal to rapid HbA1c reduction and baseline DR/insulin use; large 2025–2026 datasets generally neutral for treatment-requiring outcomes.	Risk-stratify and monitor: consider baseline DR severity and expected HbA1c drop; coordinate closer retinal follow-up early after initiation/escalation in higher-risk eyes.
Liraglutide	Marso et al. (2016) [86]	No robust DR complication signal comparable to SUSTAIN-6.	LEADER did not generate a comparable DR safety signal; early worsening risk, when present, is generally attributed to the glycemic trajectory.	Standard DR care; apply the same early worsening caution when large, rapid HbA1c reductions are expected.
Dulaglutide	Gerstein et al. (2019) [87]	Overall neutral in available evidence.	REWIND did not produce a consistent DR safety concern; comparative effectiveness analysis suggests no excess risk compared to other GLP-1RAs.	Standard DR care.
Exenatide	Holman et al. (2017) [88]	Limited contemporary DR-specific data; no strong signal in comparative analyses.	EXSCEL and comparative effectiveness evidence do not show a clear signal for sight-threatening outcomes.	Standard DR care.
Tirzepatide	Buckley et al. (2025) [89]; Popovic et al. (2024) [90]	Evolving: both an early worsening signal in a targeted cohort and protective/neutral signals in large U.S. cohorts; trial programs often excluded advanced retinopathy.	Real-world cohort suggests increased odds of incident PDR in higher-risk baseline eyes; 2026 cohorts show reduced incidence/worsening and fewer interventions vs. comparators; SURPASS protocols excluded proliferative/severe retinopathy.	Enhanced vigilance in patients with established DR and large expected HbA1c reductions; interpret risk in light of baseline eye status and comparator choice.

**Table 2:** Summary of key studies evaluating the association between GLP-1RAs and NAION or other optic disc disorders.

Disease	First Author	Methods	Main Findings
NAION	Hathaway et al. (2024) [11]	Retrospective matched cohort study using a single-center neuro-ophthalmology registry; semaglutide-exposed patients with T2DM or overweight/obesity were compared with non-GLP-1RA medication users.	Over 36 months, semaglutide exposure was associated with higher NAION risk in patients with type 2 diabetes (HR 4.28, 95% CI 1.62–11.29) and in overweight/obese patients (HR 7.64, 95% CI 2.21–26.36).
NAION	Chou et al. (2025) [101]	Retrospective multinational population-based study using a deidentified global EMR database from 160 health care organizations in 21 countries; semaglutide compared with non-GLP-1RA glucose-lowering or weight-loss medications; 1:1 propensity matching; outcomes at 1, 2, and 3 years.	Semaglutide was not associated with increased NAION risk in T2DM-only, obesity-only, or combined T2DM/obesity cohorts across follow-up time points.
NAION or ischemic optic neuropathy	Abbass et al. (2025) [102]	Retrospective matched cohort study using the TriNetX United States collaborative network; patients ≥ 12 years with T2DM or high BMI, with at least one ophthalmology or neurology visit; semaglutide or any GLP-1RA vs. non-GLP-1RA medications; 1:1 matching.	No significant increase in NAION or ischemic optic neuropathy was found in T2DM or high-BMI cohorts; in T2DM patients prescribed semaglutide, 5-year NAION RR was 0.70 (95% CI 0.523–0.937).
NAION	Simonsen et al. (2025) [96]	Danish-Norwegian national registry cohort comparing semaglutide initiators with SGLT2 inhibitor initiators in type 2 diabetes; supplementary self-controlled analyses were also performed.	Semaglutide use was associated with an approximately two- to threefold increase in NAION risk in pooled analyses; the absolute risk remained low.
NAION	Cai et al. (2025) [73]	Retrospective network study across 14 OHDSI databases in adults with type 2 diabetes using semaglutide, other GLP-1RAs, or non-GLP-1RA comparators; active-comparator new-user cohort analyses plus self-controlled case series.	The association was modest and inconsistent across analyses: hazard ratios generally ranged from about 1.4 to 2.3 depending on the comparator and NAION definition, with the authors concluding that the signal was smaller than originally reported and not uniformly elevated.
NAION risk	Hsu et al. (2025) [98]	Cohort study using the TriNetX database (2019–2023); patients with diabetes and no prior NAION prescribed semaglutide were compared with diabetic patients prescribed non-GLP-1RA antidiabetic medications.	There was no clear increase at 1 month, 3 months, 6 months, or 1 year, but semaglutide was associated with higher NAION risk at 2 years (HR 2.39, 95% CI 1.37–4.18), 3 years (HR 2.44, 95% CI 1.44–4.12), and 4 years (HR 2.05, 95% CI 1.26–3.34).
NAION	Ahmadi et al. (2025) [103]	Case series of patients developing anterior ischemic optic neuropathy while receiving semaglutide.	All four cases were male; 3 of 4 developed NAION within <1 year of treatment, and crowded discs/small Bruch’s membrane opening diameters were common. These findings suggest a potentially susceptible optic nerve phenotype but do not establish drug-induced NAION.
NAION	Lixi et al. (2025) [104]	Single case report of a 47-year-old woman without known diabetes, hypertension, or ischemic heart disease, treated first with liraglutide and then semaglutide for weight loss.	The patient developed progressive unilateral NAION temporally associated with GLP-1RA therapy. As a single case report, the observation remains hypothesis-generating and cannot distinguish a drug-related effect from individual anatomical or vascular susceptibility.
Optic nerve/ retinal adverse events	Lakhani et al. (2025) [105]	Global pharmacovigilance study using FAERS and WHO VigiBase, covering semaglutide and tirzepatide through September 2024.	Semaglutide showed increased reporting odds for ischemic optic neuropathy (FAERS ROR 11.12; VigiBase ROR 68.58) and for several retinal/vitreous adverse events, but these data do not establish incidence or causality.
NAION	Barnett et al. (2026) [110]	Retrospective cohort analysis using the TriNetX Global Collaborative Network; patients with type 2 diabetes and GLP-1 exposure vs. type 2 diabetes without GLP-1 exposure; 1:1 propensity-score matching on 20 covariates; prior NAION excluded.	After matching (388,333 per cohort), GLP-1 exposure was associated with a small but statistically significant increase in 5-year NAION risk (risk difference 0.022%, 95% CI 0.01–0.034%; RR 1.339, 95% CI 1.137–1.577).
NAION	Noh et al. (2026) [100]	Active-comparator, new-user cohort emulating a pragmatic target trial using the UK CPRD; adults with type 2 diabetes initiating a GLP-1RA or DPP-4 inhibitor; propensity-score fine-stratification weighting.	At 1 year, GLP-1RA initiation was associated with increased NAION risk (RR 2.56, 95% CI 1.44–4.86; RD 11.3 per 100,000), with a higher risk during the first 6 months and in selected subgroups.
NAION	Heberer et al. (2026) [99]	Nationwide Veterans Health Administration target-trial emulation among US veterans with type 2 diabetes on metformin and no prior GLP-1RA/SGLT2i use; semaglutide initiators compared with SGLT2i initiators using overlap weighting.	In 102,361 veterans, semaglutide initiation was associated with a higher NAION incidence (123 vs. 67 per 100,000 person-years) and a 2.33-fold higher risk (HR 2.33, 95% CI 1.54–3.54), although the absolute risk remained low.
NAION	Nogueira et al. (2026) [107]	Meta-analysis of 15 longitudinal studies (>1.5 million patients; trials plus observational studies) evaluating GLP-1RA exposure and NAION.	GLP-1RA use was associated with higher NAION risk overall (OR 1.70, 95% CI 1.23–2.36), but the absolute NAION risk was low (0.09%) with an estimated risk difference of 0.037%.
Optic nerve / vision-threatening events	Li et al. (2026) [108]	Meta-analysis of 20 randomized controlled trials including 83,288 participants with type 2 diabetes or cardiometabolic disease.	GLP-1RAs were not associated with an increased risk of the composite optic nerve/vision-threatening endpoint (OR 1.20, 95% CI 0.73–1.97) or ischemic optic neuropathy specifically (OR 1.50, 95% CI 0.49–4.63).
NAION	Abdelaal et al. (2026) [109]	Systematic review and certainty-of-evidence meta-analysis of 6 observational studies (n = 4,831,654), with pooled effect estimates and GRADE assessment.	The pooled evidence was heterogeneous and of low certainty; pooled ORs were not statistically significant overall, and the authors emphasized the fragility of the signal and low-to-very-low certainty.
NAION	Chen et al. (2026) [113]	Systematic review and meta-analysis of studies identified from database inception to 3 June 2025; 10 research articles were included and risk of bias was assessed with the Newcastle-Ottawa Scale.	Semaglutide use was associated with a significantly increased NAION risk compared with control medications (pooled HR 2.620, 95% CI 1.808–3.795); the signal became statistically significant after 2 years of exposure.
NAION	Liu et al. (2025) [112]	Systematic review and meta-analysis including 8 RCTs (31,174 patients) and 8 observational studies (1,611,278 patients); searches included MEDLINE, Embase, CENTRAL, trial registries, and sponsor contact through March 2025.	In observational diabetes studies, the pooled NAION incidence was 26.7 vs. 18.9 per 100,000 person-years with HR 1.85 (95% CI 1.20–2.85); RCTs and weight-loss cohorts did not show clear evidence of increased risk.

**Table 3:** Summary of key studies evaluating the association between GLP-1RAs and AMD.

Disease	First Author	Methods	Main Findings
Nonexudative/exudative AMD	Allan et al. (2025) [116]	Retrospective cohort study using a US electronic health records platform; patients > 60 years with at least 5 years of ophthalmology follow-up and medication documentation; 1:1 propensity matching across GLP-1RA, metformin, insulin, statin, and aspirin groups; outcomes included nonexudative AMD and exudative AMD over 5 years and across earlier time points.	GLP-1RAs were associated with a reduced hazard of nonexudative AMD versus metformin, insulin, and statins. The reduction became especially evident after 3 years (HR 0.69 vs. metformin; 0.66 vs. insulin; 0.67 vs. statins). Exudative AMD risk was also lower versus insulin at 3 years (HR 0.70, 95% CI 0.58–0.84).
Nonexudative/exudative AMD	Allan et al. (2025) [117]	Retrospective cohort study using the TriNetX US network; adults >60 years with ≥1, 2, or 3 years of ophthalmology follow-up and medication prescription data; GLP-1RA users compared with alternate glucose-lowering and lipid-lowering medication users; propensity matching on demographics, chronic disease prevalence, and severity indicators.	GLP-1RA prescription was associated with lower risk of nonexudative AMD at 1, 2, and 3 years versus glucose-lowering comparators (HR 0.79, 0.75, 0.77). Similar protective estimates were seen versus lipid-lowering drugs at 2 and 3 years. Neovascular AMD risk was also reduced across time points, but no significant effect was seen on conversion from baseline non-neovascular AMD to neovascular AMD.
Nonexudative/exudative AMD	McLaughlin et al. (2025) [118]	Retrospective cohort study using a global health research network; matched T2DM patients on GLP-1RA therapy versus non-GLP-1RA therapy; AMD incidence assessed from year 1 to year 10; a separate matched subgroup with early/intermediate nonexudative AMD at baseline was assessed for progression to advanced AMD; risk, risk difference, and risk ratio were calculated.	Among 600,816 matched patients without prior AMD, GLP-1RA use was associated with a 15% relative risk reduction in incident nonexudative AMD (RR 0.851, 95% CI 0.801–0.905) and a 20% reduction in incident exudative AMD (RR 0.80). In 4450 matched patients with early/intermediate AMD, GLP-1RA use was associated with lower progression to advanced nonexudative AMD (RR 0.715) and to exudative AMD (RR 0.729).
Exudative AMD	Shor et al. (2025) [119]	Population-based retrospective cohort study using administrative health and demographic data from Ontario, Canada; patients aged ≥66 years with diabetes and at least 12 months of follow-up after diabetes diagnosis; exposure defined as GLP-1RA use for ≥6 months; 1:2 matched cohort including 46,334 exposed and 92,668 unexposed patients; primary endpoint was incident nAMD over 3 years.	GLP-1RA exposure was associated with a higher risk of incident nAMD. Incidence was 0.2% in exposed patients versus 0.1% in unexposed patients; adjusted HR 2.21 (95% CI 1.65–2.96).
AMD in nondiabetic patients with obesity/overweight	Ahuja et al. (2025) [121]	Retrospective cohort study using the multicenter TriNetX Global Collaborative Network; patients aged ≥55 years with overweight/obesity but without diabetes; GLP-1RA users compared with users of other weight-loss drugs; propensity score matching used for primary and secondary analyses; primary outcome was incident nonexudative AMD at 5, 7, and 10 years, and secondary outcome was progression to exudative AMD.	GLP-1RAs use was associated with reduced risk of nonexudative AMD compared with other weight-loss drugs at 5 years (RR 0.16), 7 years (RR 0.13), and 10 years (RR 0.09). No significant difference was found in progression to exudative AMD.

**Table 4:** Summary of available studies on glaucoma and OHT in individuals treated with GLP-1RAs.

Ophthalmic Condition	First Author	Study Methods	Main Findings
Primary open-angle glaucoma (POAG), ocular hypertension (OHT), glaucoma treatment initiation	Muayad et al. (2025) [124]	Retrospective comparative EHR study in patients with type 2 diabetes; GLP-1RA users were compared with metformin users after propensity-score matching, with outcomes assessed at 1, 2, and 3 years.	GLP-1RA exposure was associated with lower recorded risk of POAG at 1, 2, and 3 years (RR 0.59, 0.50, and 0.59), lower OHT risk across the same timepoints, and lower initiation of first-line glaucoma treatment.
POAG and OHT in non-diabetic patients with overweight/obesity	Vasu et al. (2025) [126]	Retrospective TriNetX cohort study in patients without diabetes but with overweight/obesity; GLP-1RA users were compared with users of alternative weight-loss medications after propensity matching; outcomes evaluated at 3 and 5 years.	GLP-1RAs use was associated with lower risk of POAG and OHT at both 3 and 5 years compared with alternative weight-loss therapy.
Chronic age-related ocular disease including POAG and OHT	Allan et al. (2025) [116]	Retrospective EHR-based comparative effectiveness study assessing multiple ocular outcomes across medication groups, including GLP-1RAs versus metformin, insulin, statins, and aspirin.	GLP-1RA exposure was associated with a reduced hazard of POAG, particularly versus insulin after 3 years, whereas no consistent long-term association was observed for OHT across comparators.
POAG, OHT, and glaucoma treatment need in T2DM	Hong et al. (2026) [125]	Retrospective matched-cohort study in type 2 diabetes comparing tirzepatide with selective GLP-1RAs in a nationwide EHR network.	Tirzepatide was associated with lower risk of POAG (RR 0.50), OHT (RR 0.59), and need for first-line glaucoma treatment (RR 0.54) compared with selective GLP-1RAs.
Ocular hypertensive glaucoma (preclinical rat model)	Mouhammad et al. (2025) [123]	Experimental study in a rat model of ocular hypertensive glaucoma induced by paramagnetic bead injection; animals received systemic semaglutide and were assessed for IOP, vascular, inflammatory, and retinal ganglion cell outcomes.	Semaglutide delayed IOP elevation and showed mild vasoprotective and anti-neuroinflammatory effects, but did not improve retinal ganglion cell survival, suggesting only partial or indirect neuroprotection.
Glaucoma incidence	Amaral et al. (2025) [128]	Systematic review and meta-analysis of 5 retrospective studies evaluating glaucoma incidence in GLP-1RA users.	The primary random-effects model showed a non-significant reduction in glaucoma incidence; the association became significant in leave-one-out sensitivity analysis, indicating that the pooled result was sensitive to study composition and heterogeneity.
Glaucoma incidence in type 2 diabetes	Asif et al. (2025) [127]	Systematic review and meta-analysis focused on patients with type 2 diabetes treated with GLP-1RAs versus other antihyperglycemic agents.	The primary pooled analysis suggested a non-significant reduction in glaucoma incidence, with substantial heterogeneity, supporting a possible protective association but not a definitive conclusion.

## Data Availability

No new data were created or analyzed in this study.
